# Genetic Architecture of a Rice Nested Association Mapping Population

**DOI:** 10.1534/g3.117.041608

**Published:** 2017-04-24

**Authors:** Christopher A. Fragoso, Maria Moreno, Zuoheng Wang, Christopher Heffelfinger, Lady J. Arbelaez, John A. Aguirre, Natalia Franco, Luz E. Romero, Karine Labadie, Hongyu Zhao, Stephen L. Dellaporta, Mathias Lorieux

**Affiliations:** * Program in Computational Biology and Bioinformatics, Yale University, New Haven, Connecticut 06511; † Department of Molecular, Cellular, and Developmental Biology, Yale University, New Haven, Connecticut 06511; ‡ Department of Biostatistics, Yale University, New Haven, Connecticut 06511; § Rice Genetics and Genomics Laboratory, International Center for Tropical Agriculture, Cali 6713, Colombia; ** Commissariat à L’énergie Atomique et aux Énergies Alternatives, Institut de Génomique, Genoscope, 91000 Evry, France; †† Diversité, Adaptation, Développement des Plantes Research Unit, Institut de Recherche pour le Développement, F-34394 Montpellier, France

**Keywords:** QTL mapping, computational biology, genetic maps, nested association mapping, plant genomics

## Abstract

Describing the genetic diversity in the gene pool of crops will provide breeders with novel resources for varietal improvement. Nested Association Mapping (NAM) populations are uniquely suited for characterizing parental diversity through the shuffling and fixation of parental haplotypes. Here, we describe a set of 1879 rice NAM lines created through the selfing and single-seed descent of F_1_ hybrids derived from elite IR64 *indica* crossed with 10 diverse *tropical japonica* lines. Genotyping data indicated *tropical japonica* alleles were captured at every queried locus despite the presence of segregation distortion factors. Several distortion loci were mapped, both shared and unique, among the 10 populations. Using two-point and multi-point genetic map calculations, our datasets achieved the ∼1500 cM expected map size in rice. Finally, we highlighted the utility of the NAM lines for QTL mapping, including joint analysis across the 10 populations, by confirming known QTL locations for the trait days to heading.

Currently, one in eight people is estimated to be suffering from malnourishment, primarily in developing countries (Tscharntke *et al.* 2012). Advances in plant breeding and biotechnology, including increasing the quality and diversity of germplasm, will play an important role in reducing malnourishment and improving food security (Tester and Langridge 2010; Brennan and Malabayabas 2011). Domesticated rice is the staple of over half of the global population, comprising 50% of the daily caloric intake of 560 million Asians experiencing undernourishment (Mohanty 2013; Muthayya *et al.* 2014). The future of food security, including rice agriculture, faces incredible challenges in the form of climate change, population growth, and increasing standard of living (Godfray *et al.* 2010). For example, many areas of lowland tropical Asia suffer from erratic flooding, a trend only to increase with climate change. The introduction of submergence-tolerance into rice varieties vastly improved rice agriculture in many impoverished areas prone to flooding, reaching 3.8 million Asian farmers within 3 yr of its introduction (Ismail *et al.* 2013). This is a prime example of how identifying new sources of valuable agronomic traits can improve germplasm and, therefore, food security.

Domesticated Asian rice, *Oryza sativa*, has been subjected to distinct trajectories of domestication and cultivation, resulting in separate reservoirs of genetic diversity. Domestication of the two major clades of rice, the *japonica* and *indica* subspecies, began ∼9000 yr ago, in the river valleys of East Asia and (concurrently or possibly afterward) across the foothills of the Himalayas (Khush 1997; Garris *et al.* 2005; Londo *et al.* 2006; Kovach *et al.* 2007; Lin *et al.* 2007; Sweeney and McCouch 2007; Izawa 2008; Callaway 2014; Civáň *et al.* 2015). *Tropical japonica*, a *japonica* group, is descended from *japonicas* brought south to tropical Asia and Indonesia, whereas *temperate japonicas* were adapted to temperate Asia (Khush 1997; Kovach *et al.* 2007). *Indica* rice can be divided into *indica* and *aus* groups, although there is evidence that *aus* is descended from a separate wild population (Civáň *et al.* 2015). However, the divergence between *indica* and *japonica* predates domestication, as each subspecies resulted from sampling separate wild populations that diverged 0.44 MYA (Ma and Bennetzen 2004; Kovach *et al.* 2007; Schatz *et al.* 2014). As a result, tropical rice diversity has at least two major sources, one found in the *indicas* and the other in *japonicas*. Therefore, creating admixture populations between *indica* and *tropical japonica* varieties may result in novel combinations of tropical agriculture-adapted alleles. Moreover, traits found in *japonicas*, especially in *tropical japonicas*, such as blast resistance, robust panicles, extensive root architecture, and fewer yet sturdier stems, are just some of the features that could be beneficial in the development of improved *indica* rice varieties (Guiderdoni *et al.* 1992; Peng *et al.* 1999).

A central goal of mapping populations in plants has been to identify the genetic architecture of agronomic traits present in diverse germplasm. Mapping populations can be used to create novel combinations of parental alleles, fix parental alleles, break apart haplotypes, and to test additive or dominance effects. NAM populations, in particular, feature the development of a series of parallel Recombinant Inbred Lines (RILs), each representing a different “diversity donor” parent crossed with a common “reference” parent (Yu *et al.* 2008; McMullen *et al.* 2009). Initial F_1_ crosses are self-pollinated by single seed descent for several generations to nearly reach complete homozygosity. Despite extensive recombination, parental alleles and haplotypes become fixed. The power of this population design is through the presence of two sources of recombination: (1) the shuffling of parental alleles over several generations through segregation and genetic recombination, and (2) historical recombination of haplotypes present in the various diversity donors. This combination allows for joint mapping of traits across multiple NAM-RILs, greatly increasing the accuracy and precision of QTL discovery, especially when combined with high-density genotyping. Detection power is increased when weak QTL signals in NAM lines (possibly representing QTL with small genetic effects) are accumulated from several populations into a stronger signal. Statistical methods such as joint stepwise regression (Buckler *et al.* 2009; Ogut *et al.* 2015) and Fisher’s method (Peirce *et al.* 2007; Broman and Sen 2009) have been used for joint mapping and pooling test statistics from population-specific QTL analyses.

NAM has been designed primarily for maize (Yu *et al.* 2008; McMullen *et al.* 2009; Li *et al.* 2016), but also in other cereals such as wheat (Bajgain *et al.* 2016) and barley (Maurer *et al.* 2015). This approach has resulted in the mapping of QTL for traits such as flowering time (Buckler *et al.* 2009) and leaf blight resistance in maize (Kump *et al.* 2011; Poland *et al.* 2011), stem rust resistance in wheat (Bajgain *et al.* 2016), and flowering time in barley (Maurer *et al.* 2015).

Moreover, NAM lines may also function as an archive for genetic diversity. In the maize NAM populations, major heterotic groups in the United States and China have been represented by the careful selection of founding parental lines (Yu *et al.* 2008; McMullen *et al.* 2009; Li *et al.* 2016). The NAM populations generated from these founding lines are a resource for maize breeders to identify genetic diversity that has not yet been applied in breeding programs.

In this current study, we developed and characterized rice NAM lines to facilitate the identification of beneficial *tropical japonica* diversity, and incorporated these traits via an elite *indica* bridge variety (IR64) for future breeding initiatives. Ten *tropical japonica* diversity donor parentals were selected to be crossed with IR64 *indica* and create 10 recombinant inbred populations of ∼200 lines each for a total of 1879 NAM lines. The allelic segregation and recombination patterns in the 10 populations were described to aid future trait mapping and breeding endeavors. As a demonstration of the usefulness of our populations for mapping traits, we explored the genetic architecture of a well-characterized agronomic trait and show, through joint analysis, that combining QTL test statistics from multiple populations can provide additional mapping precision in rice NAM lines.

## Materials and Methods

### Generation of the NAM population

**Figure 1:**
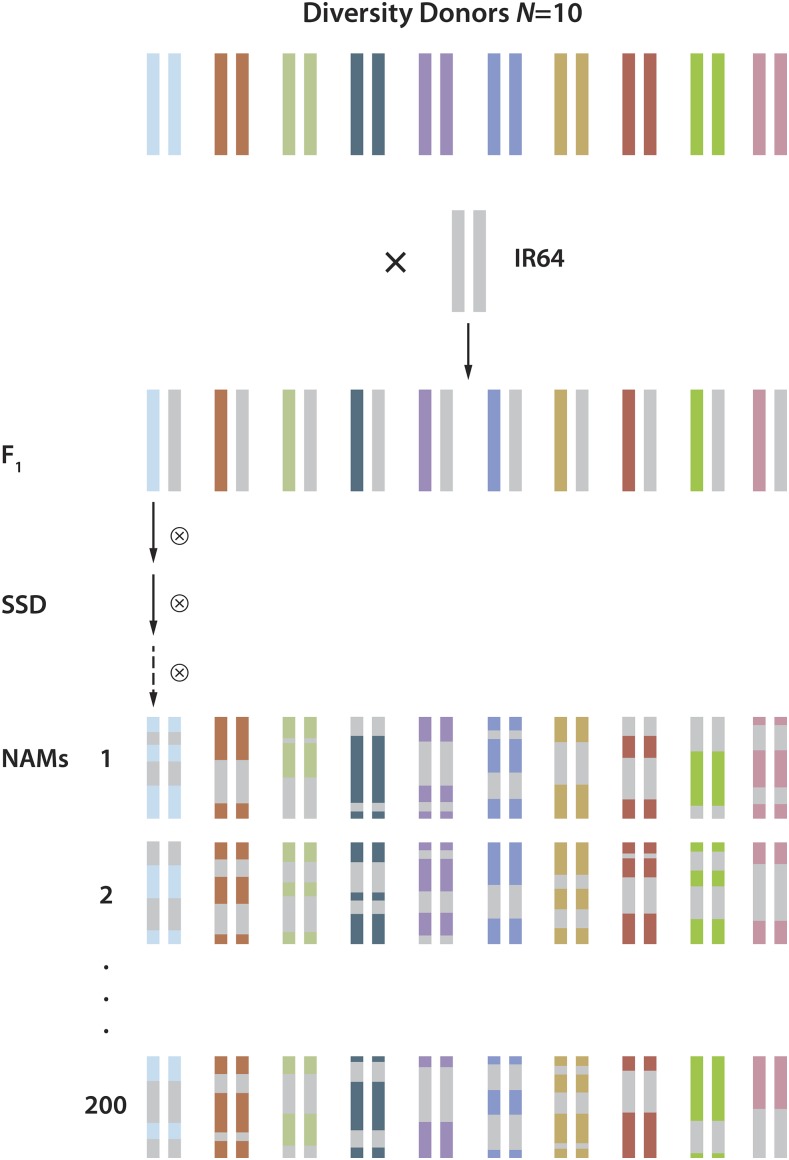
Nested Association Mapping (NAM) population design. The greater NAM population is comprised of 10 separate Recombinant Inbred Line (RIL) populations, with each population being derived from a cross between a diversity donor parent and an IR64 *indica* parent common to all 10 populations. Each RIL population consists of roughly 200 replicates of the initial cross. Therefore, there were 1879 total lines in the greater NAM population (∼200 lines × 10 RIL populations). After the F_1_, each line was self-pollinated for nine generations (IR64 × Azucena) or six generations (all other crosses) by single-seed descent (SSD). Selfing by SSD facilitates the shuffling of parental haplotypes through recombination and the fixation of haplotypes through homozygosity. Each selfing generation reduces heterozygosity by half, so a F_7_ NAM line would yield a heterozygous proportion of 0.5^6^ = 0.016. This figure is adapted from Yu *et al.* (2008).

A NAM population using *tropical japonica* diversity donors was constructed according to the protocol of the original NAM design for maize (Yu *et al.* 2008; McMullen *et al.* 2009). IR64, an elite International Rice Research Institute (IRRI) *indica* line with a complex pedigree, was selected to be the common parent and a representative of the *indica* subspecies (Figure 1). The diversity donor parentals were selected (1) for resistance and physiological traits and (2) to represent the genetic diversity present in *tropical japonica*. Azucena was chosen in particular for plant physiological traits such as days to heading; panicle and root architecture; grain traits such as aroma, shape, size, and zinc and iron content; tolerance to drought and aluminum; and resistance to *Striga*, *Xanthomonas oryzae pv. oryzae*, *Rice yellow mottle virus*, and *Magnaporthe grisea*. The IR64 × Azucena population, developed by Institut de Recherche pour le Développement (IRD), France, has been previously genotyped and described by Simple Sequence Repeats (SSRs) (Bourgis *et al.* 2008; Djedatin *et al.* 2016) and Genotype-by-Sequencing (GBS) (Spindel *et al.* 2013). The other nine diversity donors were chosen to complement mapping of the above traits in IR64 × Azucena crosses, and to expand upon the polymorphism between IR64 and Azucena. These additional diversity donors were selected from a phylogeny generated from microsatellite genotyping of representative *tropical japonica* lines in the International Center of Tropical Agriculture (CIAT) rice collection exhibiting drought resistance (E. Torres, unpublished data). *Tropical japonica* parentals were then chosen to represent as many individual clades of this phylogeny as possible.

Each of the 10 *tropical japonica* diversity donors were crossed with the common *indica* parent IR64. About three hundred F_2_ individuals were derived from each F_1_ cross. Whenever possible, all 300 samples were taken from a single F_1_ plant, with additional F_1_ plants used only when necessary. Approximately 3000 F_2_ plants were advanced to the F_7_ generation by single-seed descent, and to the F_10_ generation for the IR64 × Azucena population. Due to environmental conditions in the field and to partial genetic sterility in *indica* × *japonica*-wide crosses, some of the plants exhibited reduced fertility at different generations. As a result, just under 200 NAM lines were created for each lineage (Table 1). In total, 1879 NAM plants were generated. Outcrossing was minimized in each generation by covering inflorescences with pollination bags prior to anthesis. Table 1 indicates the population numbers for each of the 10 sets of RIL populations that were created to generate the larger NAM population.

**Table 1:** Descriptive statistics of 10 IR64 *indica* × *tropical japonica* diversity donor recombinant inbred populations

Diversity Donor of Population	Coverage per Filtered Variant*^a^*	Filtered Variants	Parental Imputed Filtered Variants	Imputed Variants	Proportion LB-Impute Imputed	Average Heterozygosity*^b^*	Proportion IR64*^c^*	Proportion Diversity Donor*^d^*	Pre-BP-Impute Lines	Post-BP-Impute Lines*^e^*	Post-BP-Impute Variants*^f^*
Azucena	2.07	7,192,750	7,246,855	15,327,750	0.99	0	0.52	0.47	187	184	15,013,664
ITA164	2.03	5,895,106	5,959,220	12,230,391	0.99	0.02	0.55	0.41	184	180	11,970,900
CT10035-42-4-4-M	2.00	7,595,531	7,597,275	15,452,515	0.98	0.03	0.52	0.44	188	178	14,656,876
CT10006-7-2-M-2	2.56	7,311,473	7,341,604	13,657,828	0.99	0.02	0.54	0.43	190	186	13,295,466
CT10037-56-6-M-M	2.03	6,718,342	6,656,774	13,920,242	0.99	0.02	0.53	0.44	188	181	13,302,052
CT10045-5-5-M-1	2.35	9,076,018	9,055,981	16,094,325	0.99	0.03	0.5	0.46	188	179	15,218,938
CT10005-12-1-M-4	2.23	8,488,910	8,469,843	15,703,353	0.99	0.02	0.51	0.46	188	184	15,284,880
CT9998-41-12-M-4	2.07	4,410,505	4,332,191	8,961,315	0.99	0.03	0.51	0.45	188	179	8,519,684
CT8556-37-1-3-1-M	1.94	4,394,706	2,602,520	5,454,213	0.98	0.03	0.52	0.43	188	177	5,126,982
CT10035-26-4-2-M	2.25	7,061,987	7,014,029	13,684,889	0.99	0.05	0.52	0.42	190	177	11,715,630

a SD ranged from 0.28 to 1.54.

b SD was << 0.001. Expected heterozygosity maximum ≤ (0.5^6^ = 1.6%).

c SD ranged from 0.10 to 0.12.

d SD ranged from 0.10 to 0.12.

e Lines ≥ 0.1 heterozygous removed.

f No missing variants in any line.

### GBS libraries

Modified flexible and scalable GBS (fsGBS) libraries were prepared according a published protocol (Heffelfinger *et al.* 2014). The Heffelfinger fsGBS protocol, which uses blunt-end restriction enzymes and employs standard Illumina Y-adapters, facilitates greater multiplexing through dual-indexed barcodes and discourages concatamer formation. Approximately 200 ng of genomic DNA was digested with *Rsa*I, a 4 bp restriction enzyme, to achieve high marker density and maximize the number of restriction fragments for Illumina sequencing. Illumina libraries were paired-end sequenced at the Yale Center for Genome Analysis. Based on read coverage, a subset of samples was selected for additional resequencing to achieve a minimum coverage threshold for all NAM lines.

### Genomics dataset protocol

**Figure 2:**
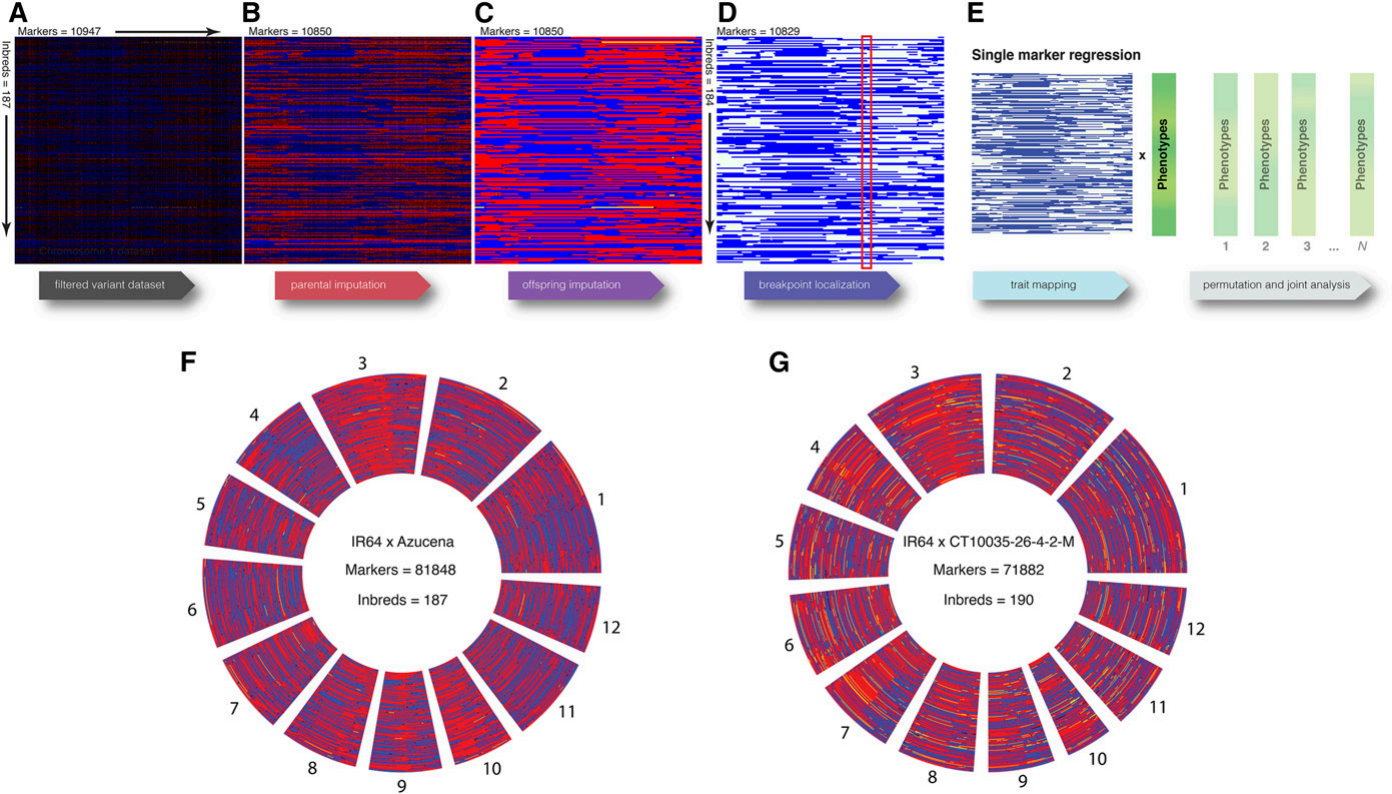
Stages of processing GBS variant data. After variant calling with GATK, variant calling data (vcf format) undergoes four stages of processing. (A–D) represent chromosome 1 of the IR64 × Azucena population, with each row representing each NAM line, and each column a marker. Red markers are homozygous for the IR64 state, blue for Azucena, yellow heterozygous, and black missing. The first stage (A) involves variant filtering with custom software described by Heffelfinger *et al.* (2014), (B) parental imputation and filtering with LB-Impute, and (C) offspring imputation with LB-Impute. Since LB-Impute may leave variants adjacent to transitions in parental state as missing, BP-Impute is applied in (D) to infer the most likely breakpoint location (red box). Genotypes may be left as probabilities or can have discrete genotypes assigned through least squares. BP-Impute is designed to prepare datasets for joint trait mapping and genetic map construction; NAM lines with excessive heterozygosity are also removed. In (E) phenotypes are regressed on each marker, then permuted phenotypes are also used to calculate locus-specific *P* values. The locus-specific *P* values can be used in joint analysis. (F) and (G) represent the entire LB-Impute dataset for the IR64 × Azucena and IR64 × CT10035-26-4-2-M NAMs, with each concentric circle depicting a separate NAM genome. For (G), blue is the homozygous CT10035-26-4-2-M state. GBS, genotype-by-sequencing; NAM, Nested Association Mapping.

GBS paired-end reads were aligned with Novoalign (Hercus 2012) to version 7 of the Nipponbare reference genome (Ouyang *et al.* 2007). Variant calling was performed with GATK (McKenna *et al.* 2010; DePristo *et al.* 2011; Auwera *et al.* 2013), and a custom algorithm was used for filtering raw variant calls and filtering imputation results, as described in Heffelfinger *et al.* (2014). Variant calling and imputation was performed on a population-by-population basis (Figure 2). LB-Impute (Fragoso *et al.* 2016) was first used to impute missing parental markers that were sequenced in the offspring. A second round of parental variant filtering was executed to remove any heterozygous parental markers and to confirm that each marker was polymorphic between the parentals. Next, offspring imputation was performed based on the imputed parental genotypes (parameters for all of the above programs are described in Supplemental Material, Note S1 in File S1). LB-Impute was chosen for its ability to perform parental imputation and for its high imputation accuracy in regions of low-coverage residual heterozygosity. The final imputed dataset represents high-confidence markers present in each population with low-confidence markers left as missing.

The missing, low-confidence markers were often present in transition regions of recombination breakpoints. Therefore, a second algorithm, Breakpoint Imputation (BP-Impute, Note S2 in File S1), was developed to improve the precision of genotyping in regions containing recombination breakpoints. BP-Impute calculates a Markov chain across the missing regions, with chains in either direction constrained to the flanking parental state. The transition probability was the proportion of recombinant genotypes across each interval. Emission probabilities were the binomial probabilities of emitting genotypes given read coverage and the constrained parental state. The probabilities from both chains were normalized to sum to one, and the weighted average genotype is thus summed from these probabilities.

In each BP-Impute population dataset, a limited number of lines with excess heterozygosity (> 10%), representing potential outcrossing, were removed so that estimations of recombination would be more representative of the rest of the population. The resulting dataset was comprised of genotype probabilities, with each data point representing the probability of a genotype being homozygous for the diversity donor allele. The genotype probabilities were used for measuring segregation distortion and trait mapping. For measuring local recombination rates and the genetic map, discrete genotypes were assigned to genotype probabilities by applying least squares through a separate, custom R script (BP-Impute, Note S2 in File S1).

### Parental sequencing, clade assignment, and allele frequency analysis

The 11 parental lines were sequenced as part of the International Rice Genomic Initiative (IRIGIN), a France Genomique project by whole-genome Illumina paired-end sequencing at an average coverage of 35 ×. Whole-genome sequencing (WGS) datasets of parental lines were subject to the same read alignment, variant calling, and variant filtering as the GBS data, but with slightly different parameter settings (Note S1 in File S1). The filtered WGS variant data were trimmed by randomly selecting a marker within bins of 5 kb. For this set of 72,193 markers, the SNP-Seek database (Alexandrov *et al.* 2015) was queried to extract the trimmed marker data for the (3000 Rice Genomes Project 2014). In total, 49,431 markers and 2656 lines were extracted from SNP-Seek. Only markers that were homozygous in the NAM parentals were selected from the SNP-Seek marker set in order to exclude false heterozygous regions arising from poor read mapping in repetitious genomic regions. Lines annotated as *indica*, *temperate* *japonica*, and *tropical japonica* were merged with the NAM parental dataset and were imputed using the FILLIN algorithm (Tassel 5 version 20160428) (Swarts *et al.* 2014). Then, the imputed dataset was filtered for missingness (lines and markers with missingness >0.1 were removed, then the set of complete, nonmissing markers was extracted), and pruned for linkage disequilibrium (LD) (markers in bins of 100 kb with pairwise correlation >0.3 were removed). The final merged, imputed, complete, and LD-pruned dataset consisted of 2655 lines (including the 11 NAM parentals) genotyped at 7152 markers. Principal component analysis (PCA) was performed on this dataset, using the prcomp R library, to elucidate the placement of the NAM parentals in the greater population structure of rice.

The parental contribution of IR64:DD alleles was determined for each marker in the NAM populations using the BP-Impute dataset. Statistical evidence of deviation from the 1:1 expected segregation ratio was measured with a χ^2^ goodness of fit test.

### Recombination analysis

The genetic map for each population was calculated from the LB-Impute (Fragoso *et al.* 2016) datasets (imputed with the –keep flag), with three different methods. The –keep flag was used so that both unimputed and missing variants remain in ambiguous regions surrounding recombination breakpoints. In method 1, multipoint genetic maps were calculated with Mapmaker/EXP3.0 (Lincoln *et al.* 1993). The LB-Impute datasets were filtered for unique markers, and multipoint maps were calculated with Lincoln and Lander error detection method (Lincoln and Lander 1992) both on and off. In method 2, two-point maps were estimated from full imputation of the LB-Impute data with argmax from R/QTL (Broman *et al.* 2003; Broman and Sen 2009). The argmax function was used to impute the LB-Impute datasets in order to further resolve breakpoints. The Mapmaker and R/QTL analyses were performed in MapDisto 2.0 (Lorieux 2012; Heffelfinger *et al.* 2017). These three calculations were compared with the genetic maps generated with method 3 based on BP-Impute datasets.

In method 3, the assign genotypes function was used to identify breakpoints in BP-Impute datasets and reach full imputation. Then, the genetic distance between each pair of adjacent markers was calculated through two-point analysis. The recombination rate per meiosis between adjacent markers, *r*, was calculated with the unbiased estimate for RILs self-pollinated by single seed descent, as described by Martin and Hospital, $\hat{r}=\frac{m\left(N-m-1\right)}{2{\left(N-m\right)}^{2}}$, where *m* is the number of recombinants among *N* RILs (Martin and Hospital 2006). Transitions into heterozygous states from homozygous variants, or vice versa, were either set as missing (weighted as 0, with all other transitions weighted as 1) or were weighted as 0.5 (with all other transitions weighted as 1). This was performed to examine the effect of heterozygosity on the genetic map sizes, while taking into account the theoretical fixation of heterozygous regions as homozygous after infinite selfing generations. From estimates of *r* with all methods, the Kosambi mapping function was used to measure the genetic distance between each pair of adjacent markers.

A joint genotype dataset was also created to compare the joint genetic map size with individual population genetic maps. The union of all markers in the 10 populations was taken, imputed with the R/QTL algorithm argmax function (Broman and Sen 2009) (double recombinant probability set as 0.005), then the joint two-point map was calculated with the Martin and Hospital (2006) $\hat{r}$ estimate in MapDisto 2.0 (Lorieux 2012; Heffelfinger *et al.* 2017).

The Gaussian kernel method was then applied to smooth the BP-Impute two-point genetic distances for analysis of local recombination rate. The kernel method was chosen to reduce the effects of any possible errors introduced through genotyping or breakpoint imputation. A similar analysis performed by Spindel was used as a guide (Spindel *et al.* 2013). For each measurement of genetic distance, a Gaussian (normal) density function with σ = 0.5 was centered on the physical position, in mega bases, of the corresponding marker. The kernel was set to 0 where markers were off the queried chromosome, and the density was then renormalized so it would sum to 1. Each marker pair’s two-point genetic distance was then recalculated according to the weights determined by the kernel.

### Trait mapping

The number of days to heading (the emergence of the rice inflorescences) since sowing date was selected to demonstrate the utility of the NAM design for trait mapping in individual populations, and jointly across all 10 populations. Days to heading was phenotyped at CIAT between 2011 and 2013, with sowing dates in January, February, March, May, June, July, and November. All populations were phenotyped for the trait; two lines were excluded from QTL mapping for erroneous data entries.

The genotype probability dataset from BP-Impute (each genotype represented by the probability of representing the homozygous, diversity donor state) was used for trait mapping. With single marker linear regression, each marker in each population was tested for the null hypothesis of no additive genetic effect at the locus (the *y* intercept as the best explanation of the phenotype). The alternative hypothesis was that the genotypes explain some variance of the phenotype. The *F* statistic value (henceforth referred to as the *F* value), from the R lm function, was stored for each marker. The *F* value is asymptotically equivalent to the LOD score (Broman and Sen 2009) and is convenient for its easy and rapid extraction from the lm function. Linear regression was used to fit the null and alternate hypotheses so that continuous genotype probabilities may be used. Given the saturation of the genetic map, use of interval mapping (Lander and Botstein 1989) was not required.

The methodology of Peirce regarding permutation, linear interpolation, locus-specific *P* values, and joint analysis (Peirce *et al.* 2007) was applied through custom R scripts written for trait mapping in the 10 populations. In order to facilitate downstream significance testing, the phenotypes were permuted 1000 times, and each permutation was regressed on the markers. In order to calculate locus-specific *P* values, the probability of encountering an *F* value as large as the observed value in the 1000 permutations was determined for each marker.

### Joint analysis for trait mapping and allelic frequency

For *F* value peaks jointly segregating in the QTL analysis, and regions of segregation distortion present in multiple populations, joint analysis was used to pool test statistics from the respective populations. To this end, Fisher’s combined probability test (Fisher’s method) was applied to *P* values from χ^2^ goodness of fit tests (1:1 parental allele segregation) and to locus-specific *P* values from trait mapping. According to Fisher’s method, the natural logs of *P* values were summed for each of the markers in the local joint marker set. This sum, when multiplied by −2, has a χ^2^ distribution with degrees of freedom equal to twice the number of summed log *P* values under the null hypothesis. In order to create the joint marker set for these analyses, *P* values (segregation distortion) or *F* values (trait mapping) were linearly interpolated by physical position. This allowed for each population to have a test statistic at the same set of positions representing the region of interest. Joint analysis was performed on a targeted basis at these specified regions.

For the joint trait mapping analysis, genome-wide adjusted *P* values were calculated. This refers to the proportion of pooled *P* values obtained from random permutations that are as extreme as the observed pooled value. For each of the populations subject to pooling, locus-specific *P* values were calculated for every permuted *F* value at a given marker. A locus-specific *P* value was then randomly selected at every marker, from each population, to create a random pooling. This permuted pooling was performed 1000 times, and the genome-wide adjusted *P* value was estimated by the proportion of the permuted pooled values larger than the pooled observed data. The threshold for significance was the 95th percentile of permuted poolings.

In order to create a support interval for the joint QTL peaks, the consensus support interval among population-specific 1.5-*F* value or 3-*F* value support intervals [comparable to the 1-LOD, 1.5-LOD, or 1.8-LOD interval commonly used (Peirce *et al.* 2007; Broman and Sen 2009)] were calculated. This method was used as an approximation to a true C.I. comparable across populations. A similar approach was used for support intervals surrounding segregation distortion loci, except the support intervals were only calculated for the pooled χ^2^ values, not for each individual population-specific value.

All analyses, including studies of recombination, segregation distortion, and trait mapping, were performed with custom R scripts. These programs were tested and run with R version 3.3.2 (R Core Team 2014).

### Data availability

NAM parental and offspring germplasm and genotypes are available by request. Genotypes are offered through a material transfer agreement (MTA) from Yale University via stephen.dellaporta@yale.edu and the germplasm through a MTA from the International Center of Tropical Agriculture, via the corresponding author. LB-Impute (Fragoso *et al.* 2016) and BP-Impute are available through a license on the Dellaporta Laboratory Github site https://github.com/dellaporta-laboratory and MapDisto v2 (Lorieux 2012; Heffelfinger *et al.* 2017). 

## Results

### Selection of NAM parentals

**Figure 3:**
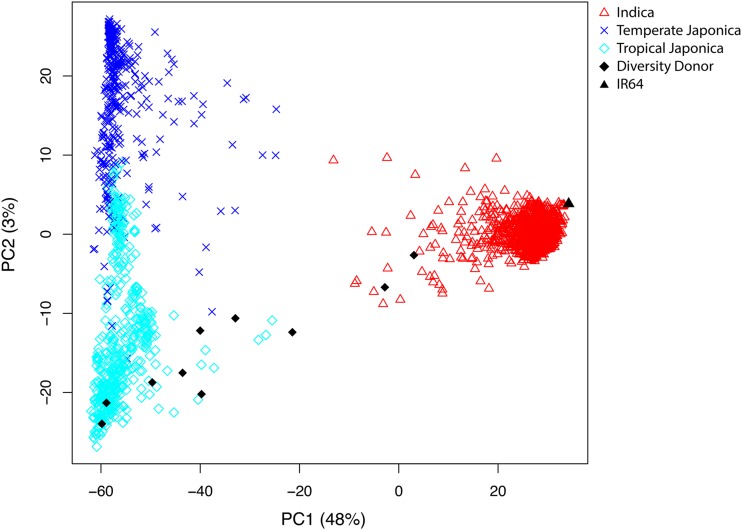
Principal Component Analysis (PCA) of 2644 *indica* and *japonica* lines and 11 Nested Association Mapping (NAM) parental lines. PCA was performed on 1789 *indica*, 371 *temperate* *japonica*, and 484 tropical *japonica* lines of the 3000 Rice Genomes Project merged with 11 NAM parental lines. The first principal component (PC), demonstrating an *indica–japonica* axis in the dataset, describes 47% of the total variance. The second PC describes the differences between the two *japonica* subtypes and 3% of the total variance. IR64, the *indica* common parent, clusters with *indica*, while the diversity donors appear on a gradient of *indica–tropical japonica* admixture. Two diversity donors in particular, CT8556-37-1-3-1-M and CT10035-26-4-2-M, cluster with *indica*, potential evidence admixture in the pedigree of these two lines. Azucena and ITA164, on the other hand, cluster tightly with *tropical* *japonica*.

The 10 *tropical japonica* diversity donors and IR64 *indica* common parent were genotyped through 35 × WGS. We explored the genetic relationship between the NAM parental lines and the general diversity found in the *indica* and *japonica* rice subspecies in the 3000 Rice Genomes Project (Rice Genomes Project 2014). PCA on this dataset revealed three clusters of rice lines, corresponding to the *indica* subspecies, and *temperate japonica* and *tropical japonica* groups of the *japonica* subspecies (Figure 3). The first principal component, representing 48% of the variance in the dataset, described the *japonica–indica* axis in rice. The second principal component, representing 3% of the variance, coincided with the separation between *temperate* and *tropical japonica*. IR64, the *indica* common parent, clustered with the other *indica* lines on the far right of the *indica* axis. Although all of the diversity donors were chosen as *tropical japonica* lines, two of these lines were located closer to *indica* along the *japonica–indica* axis (PC1). Azucena and ITA164 clustered as expected with *tropical japonica* at the far left of the *japonica* axis, whereas CT8556-37-1-3-1-M and CT10035-26-4-2-M were located within the *indica* cluster closer to the right of the axis.

### Population summary

The 1879 NAM lines were sequenced and genotyped with a customized GBS method and informatics pipeline of variant calling, variant filtering, and imputation. Ten genotype datasets were created, one for each *tropical japonica* diversity donor × IR64 *indica* recombinant inbred population. Genotyping metrics describing these populations were summarized in Table 1. Genomic DNA from the NAM lines was digested by *Rsa*I to create GBS libraries (Heffelfinger *et al.* 2014), multiplexed according to population membership, and subjected to paired-end Illumina sequencing. Multiplexed sequencing reads were next deconvoluted by molecular barcoding, aligned to the Nipponbare reference genome version 7 (Ouyang *et al.* 2007), then further processed through variant calling, variant filtering, and imputation (see *Materials and Methods* for details). The first round of imputation resulted in ∼99% of variants being imputed via LB-Impute (Fragoso *et al.* 2016); a final stage of imputation was required to genotype markers surrounding recombination breakpoints to create a complete dataset (BP-Impute, Note S2 in File S1) for each population. These complete datasets were used for trait mapping and genetic map construction.

Dense genotyping was achieved in all 10 populations (Table 1). There were 1879 total lines, ranging from 184 to 190 per population. Average coverage per filtered variant in each population ranged from 1.94 to 2.56. The range of LB-Impute imputed variants ranged from 5,454,213 (among 188 lines) to 16,094,325 (among 188 lines). The greatest average heterozygosity was in IR64 × CT10035-26-4-2-M, at 5%. BP-Impute removed any line with > 10% heterozygosity, so after this processing step, the number of lines in each of the 10 populations ranged from 177 to 186.

### Distribution of parental alleles

**Figure 4:**
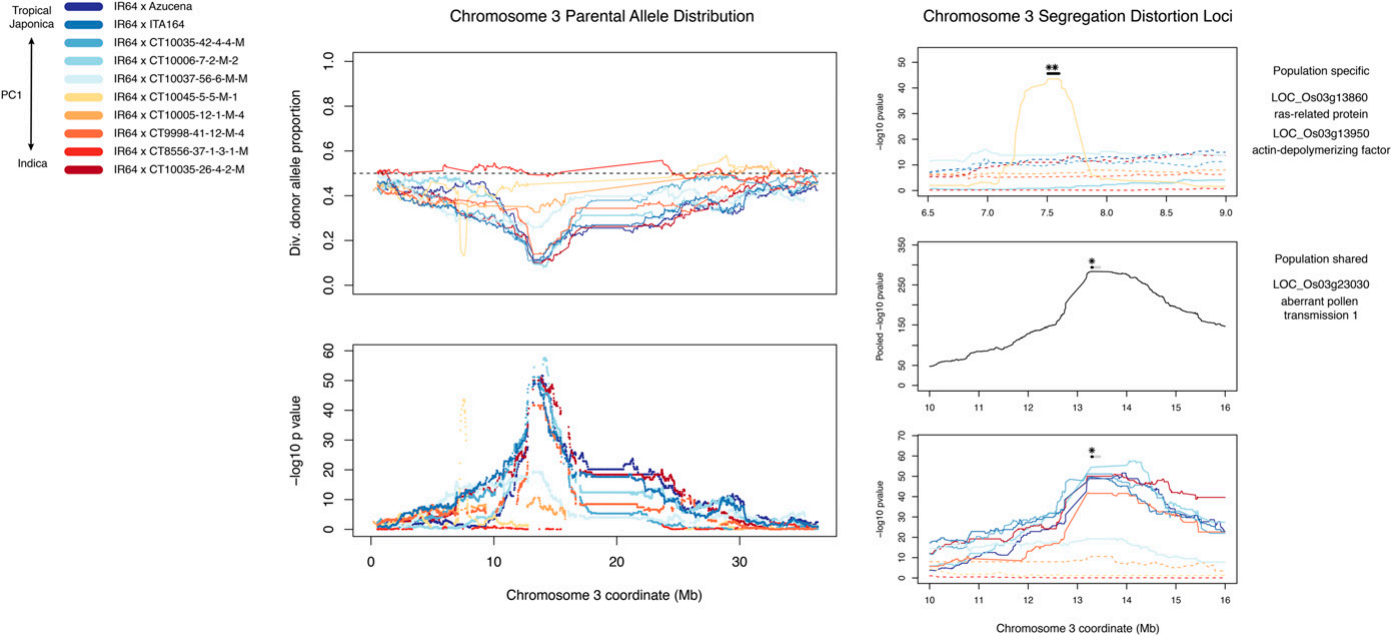
Segregation distortion in chromosome 3. Chromosome 3 exhibits strong segregation distortion, in the direction of favoring the IR64 allele, for two loci. One locus is specific to IR64 × CT10045-5-5-M-1, the other loci has a shared signal in six populations. The left panel is the color code key; a heatmap from blue to red representing the position of the respective diversity donor on the *japonica–indica* axis from principal component (PC) 1. The center panel features the diversity (div.) donor allele proportion at each marker (top) with the dashed line across 0.5 reflecting the expected segregation ratio. The bottom plot on the panel shows *P* values from a χ^2^ test of the 1:1 null hypothesis. The right panel focuses on the two loci of distortion encountered on the chromosome. Full lines represent the population(s) used in the joint analysis and dashed lines were excluded populations. For the population-shared locus, the *P* values were pooled via Fisher’s method. The black line over the peaks shows the 1.5-χ^2^ support interval for the peak, the gray line is the 3-χ^2^ support interval. Asterisks reflect potential gene candidates for distortion factors. They were chosen for their previously described role in pollen tube growth in rice or other plants. There were 16 genes in the 1.5-χ^2^ support interval for the population specific locus and two genes for that of the shared locus.

We examined whether each genomic region of the diversity parent was present in at least one member of the respective NAM subset. In all 10 NAM populations, there was no fixation of IR64 reference parent alleles at any genotyped location (Figure 4 and Figure S1 in File S1). Diversity donor alleles were present at all sites, indicating a full capture of genetic diversity from our *tropical japonica* parental lines.

We did find evidence of shared and population-specific signals of segregation distortion when examining parental contributions in the 10 NAM populations. The proportion of diversity donor to reference parent alleles deviated from the expected 1:1 in several locations. Depending on the diversity donor of a respective NAM population, some of the population exhibited shared or unique trends in the segregation ratio. There were shared signals of IR64 alleles being favored in chromosomes 3, 7, and 9. In these regions, many of the populations experience diversity donor allele proportions < 15% (Table S1 in File S1).

Applying Fisher’s method to pool *P* values from χ^2^ tests of a 1:1 segregation ratio confirmed segregation trends among the NAM populations (Figure 4 and Figure S1 and Table S1 in File S1). For populations exhibiting similar trends in segregation distortion, the natural logs of χ^2^ *P* values were summed for each of the markers in the joint marker set.

### Identification of segregation distortion loci

The strongest evidence of segregation distortion was found on chromosomes 3, 6, 7, and 9 (Table S1 in File S1). These regions all experienced distortion favoring the IR64 allele. There existed a population-specific locus on chromosome 6 and shared peaks on chromosomes 7 and 9. The minimum diversity donor allele frequencies for markers at these peaks ranged from 0.07 to 0.15. Finally, only chromosomes 1, 4, and 6 presented substantial evidence of segregation distortion in the direction of the diversity donor allele, with extended regions of distortion for one of the 10 populations.

There were two peaks of segregation distortion on chromosome 3; one was specific to IR64 × CT10045-5-5-M-1 and the other was shared between six populations (Figure 4 and Figure S1 in File S1). The 1.5 χ^2^ interval for the population-specific peak was 94,269 bp, containing 16 genes centered at 7.6 Mb. At this population-specific peak, the minimum diversity donor allele frequency at a genotyped marker was 0.13. Two genes in this interval that could potentially contribute to pollen tube growth include the *ras*-related protein LOC_Os03g13860 (Cheung and Wu 2008; Szumlanski and Nielsen 2009), and the *actin depolymerizing factor* LOC_Os03g13950 (Dong *et al.* 2001; Feng *et al.* 2006; Zhang *et al.* 2007; Li *et al.* 2010). Expanding support to 3-χ^2^ did not greatly increase the interval.

In the shared peak, the 1.5-χ^2^ support interval was 16,015 bp, containing two genes centered at 13.3 Mb. The minimum diversity donor allele frequency at the pooled peak was 0.10. One of the two genes in this interval is a rice homolog of the maize *aberrant pollen transmission 1* gene, which influences the speed of pollen tube germination in maize (Xu and Dooner 2006). Increasing the support to 3-χ^2^ expanded the support interval to 173.5 kb.

### Recombination analysis

To assess the accuracy of our genotyping and imputation methods, we surveyed the number of recombination events in each NAM line, and used this information to calculate total genetic maps for every population. Across all NAM lines, the average number of recombination events was 18.9 with a SD of 10.9 (Figure S2 in File S1). When we examined the average number of recombination events in the 10 populations, ANOVA suggested that the differences between the population means were significant, with an *F* value of 4.5 and a *P* value of 6.8 × 10^−6^. The population with the greatest average number of recombination events was IR64 × CT10006-7-2-M-2, with 21.6 events. The fewest average number was IR64 × Azucena, at 16.1 events.

**Figure 5:**
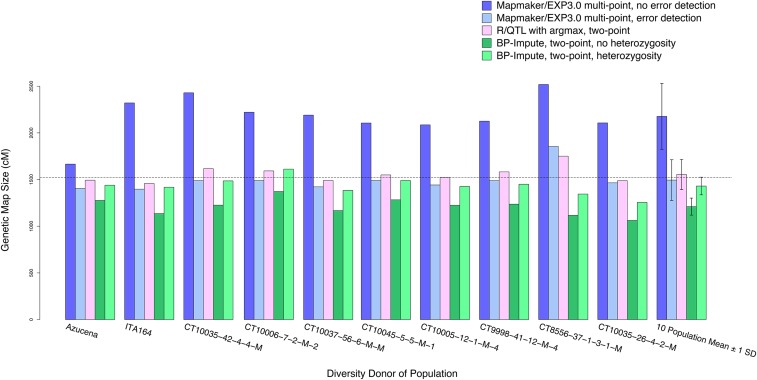
Genetic map sizes of 10 NAM populations. Genetic maps were calculated following (i) method 1, multipoint analysis with MapMaker/EXP3.0 with error detection off and on; (ii) method 2, two-point analysis with MapDisto on data imputed with the R/QTL argmax function; and (iii) method 3, two-point analysis on BP-Impute datasets, without and with integration of heterozygous–homozygous transitions. The expected map size in rice is represented by the horizontal dotted line, at 1521.6 cM (Harushima *et al.* 1998). The 10 population means are shown with ±1 SD.

Of the three genetic map construction strategies, multipoint Mapmaker/EXP3.0 (Lincoln *et al.* 1993) produced the largest maps, averaging across the 10 populations, at 2177.0 cM (± SD 352.8 cM) without error correction, and were significantly shortened with error correction, at 1494.2 cM (± SD 218.2 cM) (Figure 5). R/QTL argmax (Broman *et al.* 2003; Broman and Sen 2009) imputation produced an average two-point map size of 1554.5 cM (± SD 161.7 cM). BP-Impute resulted in 1210.2 cM (± SD 91.2 cM) without counting heterozygous–homozygous transitions and increased to 1430.8 cM (± SD 94.4 cM) with counting transitions (Table S2 in File S1). The population with the greatest map with BP-Impute and heterozygosity was IR64 × CT10006-7-2-M-2 at 1612.0 cM and the smallest was IR64 × CT10035-26-4-2-M at 1255.8 cM.

In the joint population dataset, where all NAM lines from the 10 populations were imputed together by R/QTL argmax (Broman and Sen 2009) as a single dataset, the original size of the union of markers was 170,000. This value was collapsed to 50,079 markers by removing loci with no recombination with other loci. Then, after R/QTL argmax imputation, the dataset consisted of 50,006 complete markers with no missing values. The two-point genetic map (Martin Hospital estimate for $\hat{r}$) produced a total map size of 1348.7 cM for the joint dataset (Table S3 in File S1).

**Figure 6:**
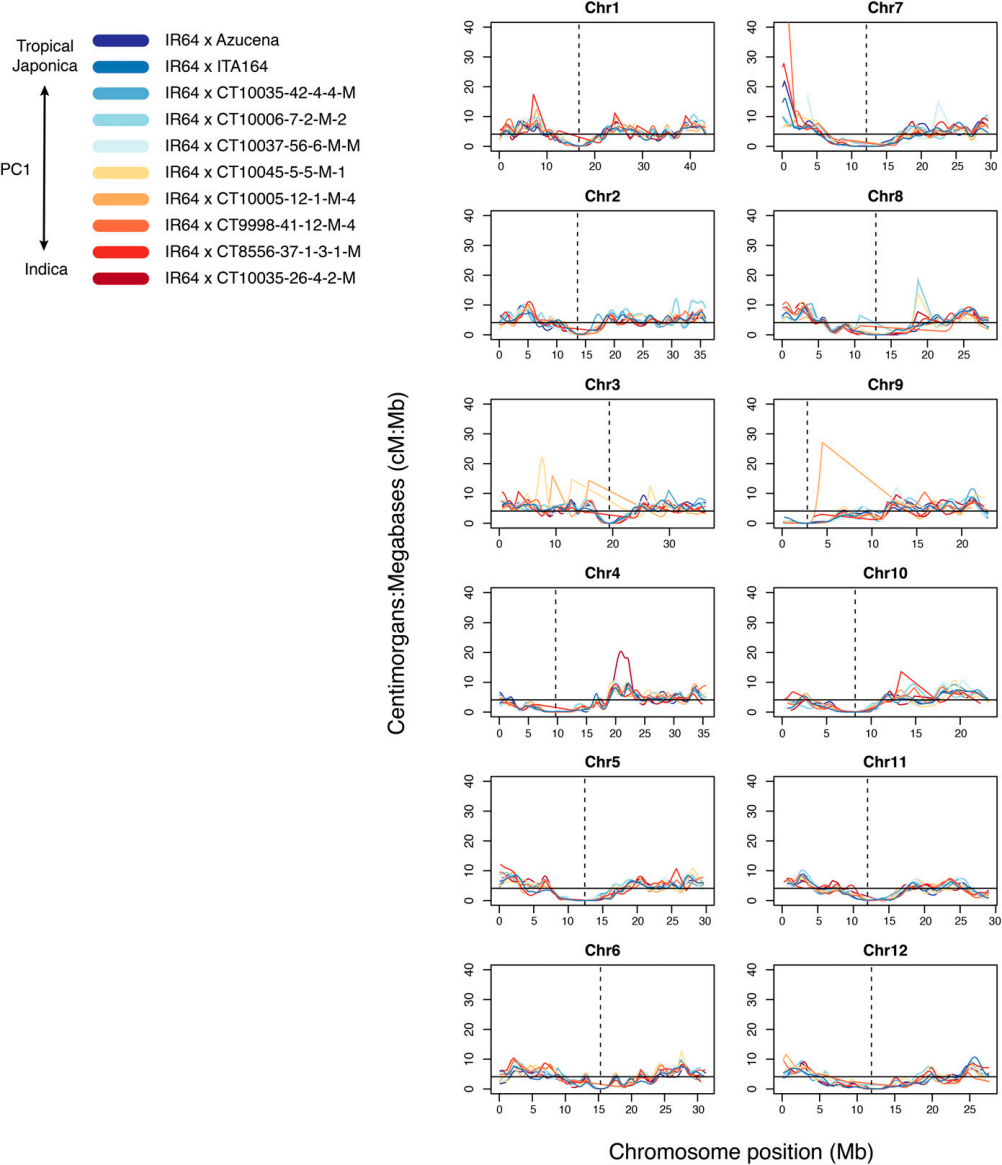
Gaussian smoothed ratio of the genetic and physical maps. Two-point genetic distances were calculated for each pair of adjacent markers, for all populations, using method 3 with integration of heterozygous–homozygous transitions and the Kosambi mapping function. A Gaussian kernel with σ = 0.5 was placed over each marker to smooth the two-point genetic and physical distances; then the ratio was taken and plotted. The horizontal line is the expected ratio, at 1 cM per 0.244 Mb or 4.1 cM:Mb, per Chen *et al.* (2002). The vertical dotted line is the approximate location of the centromere, as defined by the presence of centromere-specific retrotransposons described by Cheng *et al.* (2002). Chr, chromosome; PC, principal component.

Although the genetic maps calculated from imputed data of each population globally approximated the map size for rice, there were local variations in recombination rate. Each of the 10 populations demonstrated deviations from the expected average recombination rate [1 cM per 0.244 Mb, or 4.1 cM:Mb (Chen *et al.* 2002)], with local hotspots being both unique and shared among the NAM subsets (Figure 6). As expected, all populations experienced a decrease in recombination at the centromere (Cheng *et al.* 2002; Ouyang *et al.* 2007). However, throughout the remainder of the genome, the cM:Mb ratio remained close to the expected value of 4.1 cM:Mb (Chen *et al.* 2002).

### Joint QTL analysis

Days to heading, defined as the number of days from sowing until the emergence of inflorescences, is a complex trait of agronomic significance. The short arm of chromosome 3 features four genes, the CCCH-type zinc finger gene LOC_Os03g02160, *early heading date 4* (*Ehd4*) (Gao *et al.* 2013), MIKC-type *MADS-box 50* gene (*OsMADS50*) LOC_Os03g03070 (Lee *et al.* 2004), *DNA-binding with one finger 12* (*OsDof12*) LOC_Os03g07360 (Li *et al.* 2009), and *rice phytochrome B* (*OsPhyB*) LOC_Os03g19590 (Takano *et al.* 2005). Our goal was to determine whether a joint mapping approach in the NAM population could reveal the complex genetic architecture in this region. The entire NAM population was phenotyped for a number of traits including days to heading. The global mean of days to heading, among all NAM lines, was 91.86 d, with a SD of 6.69 d (Figure S3 in File S1). The greatest mean days to heading, for an individual population, was IR64 × CT10035-26-4-2-M at 99.90 d. The fewest mean days to heading was IR64 × Azucena at 88.82 d. ANOVA suggests that differences between the population means were statistically significant, with an *F* value of 55.00 and a *P* value < 2.2 × 10^−16^.

**Figure 7:**
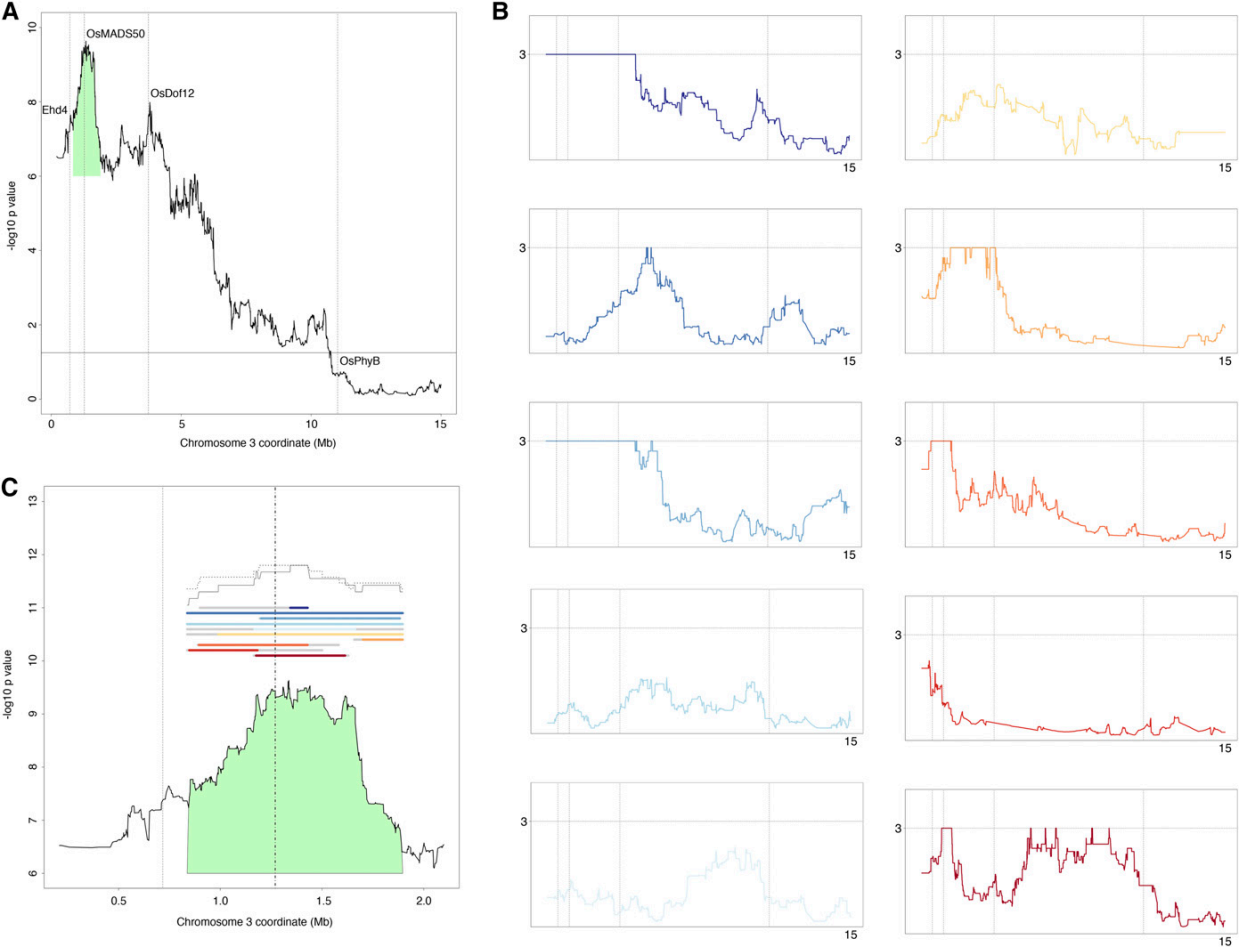
Joint and individual population QTL analysis of days to heading on the short arm of chromosome 3. (A) Locus-specific *P* values from each population were pooled through Fisher’s method. The positions of three out of four known photoperiod genes are represented by vertical lines on the *x*-axis. The horizontal line is the 95th percentile of permuted poolings. (B) The locus-specific *P* values for each population; the proportion of single marker regressions with permuted phenotypes yielding an *F* value as extreme as the observed *F* value. Since there were 1000 permutations, the minimum *P* value is 10^−3^. The *y*-axis in all subplots are the −log10 *P* values, on a linear scale, with the horizontal line reflecting the 10^−3^ minimum *P* value. The *x*-axis is the same as (A), with the vertical lines representing the four genes. (C) The peak corresponding to OsMADS50, also green in (A), is further examined in individual populations. The colored lines are the 1.5-*F* value support intervals; the 3-*F* value support intervals are in gray. The summations of the support intervals, indicating consensus between the individual support intervals, are located above. The 1.5-*F* value consensus is the solid line, and the 3-*F* value consensus is the dotted line. The vertical lines are the positions of Ehd4 and OsMADS50. The color coding in (B) and (C) for the 10 populations is consistent with the other figures, and reflects the principal component analysis in Figure 3. QTL, quantitative trait loci.

The joint analysis of days to heading QTL (Figure 7A) revealed three joint peaks that correspond closely with *Ehd4*, *OsMADS50*, and *OsDof12*. The peak that aligned with *OsMADS50* had the smallest *P* value encountered through joint permutation testing; *Ehd4* was located adjacent to a nearby subpeak of *OsMADS50*. *OsDof12*, located further downstream, aligned to the second highest peak. The *OsPhyB* gene coordinates were positioned beneath the 17.79 (−log10; 1.25) 95th percentile of permuted pooling replicates. In order to identify gene candidates, the MSU Rice Genome Annotation Project (Ouyang *et al.* 2007) was consulted for gene locus locations and the BLAST (Altschul *et al.* 1990) tool was used to search for homologs in other plant species.

Of the individual population locus-specific *P* values (Figure 7B), IR64 × Azucena and IR64 × CT10035-42-4-4-M experienced the minimum possible *P* value (10^−3^) for entire interval containing the three jointly segregating genes, *Ehd4*, *OsMADS50*, and *OsDof12*. The other populations either demonstrated weaker peaks or troughs in between the three genes. No population exhibited clear evidence for a peak at the *OsPhyB* gene.

The *F* values differed greatly among the populations (Figure S4 in File S1). IR64 × Azucena had the greatest *F* values by far, with a maximum of 69.79, and a complex QTL peak structure in the vicinity of *Ehd4* and *OsMADS50*. IR64 × CT10035-42-4-4-M had the second highest *F* values and a similar QTL peak structure. Other populations, despite demonstrating clear peaks in the permutation analysis, had much smaller *F* values as compared to IR64 × Azucena.

The joint peak corresponding to *OsMADS50* was subjected to more in depth analysis to better understand the individual contributions of each population to the joint peak (Figure 7C). The analysis interval was chosen to avoid inclusion of the minor peak near *Ehd4*. The greatest *F* value for each population, between 0.82 and 1.90 Mb on chromosome 3, was determined, and the 3 and 1.5-*F* value support interval was overlaid on the joint peak. Each set of support intervals was then summed to calculate a consensus interval. The two summations were plotted above the individual support intervals in Figure 7C, with the 1.5-*F* value as a solid line and the 3-*F* value as a dotted line. The *OsMADS50* gene is upstream of the 1.5-*F* value consensus (85,226 bp) within this interval. IR64 × Azucena was the primary driver of this consensus region. Homeobox genes and protein kinases are found in this region, including a receptor-like protein kinase LOC_Os03g03280 homologous to *CURVY1* that controls flowering time in *Arabidopsis* (MSU rice BLAST: *e* score 10^−160^, top query coverage 96.32, top Id 38.57) (Gachomo *et al.* 2014). From the 3-*F* value support intervals, the consensus region is 235,514 bp and contains *OsMADS50*.

## Discussion

We have developed and characterized the genetic structure of 10 recombinant inbred populations of rice that combined for a total of 1879 NAM lines. These populations represent a genetic resource for mapping traits relevant to tropical agriculture and for the study of the genetic properties of *indica* × *tropical japonica*-wide crosses. Through the power of joint analysis, we show that regions of complex genetic architecture or segregation distortion can be finely dissected and characterized. By applying newly developed variant filtering and imputation methods, and combined with the improved fsGBS method (Heffelfinger *et al.* 2014), our results combine both dense genotyping and accurate detection of recombination events, without the trade-off of excessive erroneous recombination events. The average map size of our BP-Impute datasets, 1430.8 cM (Table S2 in File S1), deviates 90.8 cM from the expected map size of 1521.6 cM (Harushima *et al.* 1998). A previous estimate of the genetic map of the IR64 × Azucena RIL population was 1862 cM, an overestimate of the expected map size by 331 cM (Spindel *et al.* 2013).

It is likely that the BP-Impute genetic maps (method 3) are slightly shorter (1430.8 cM ± SD 94.4 cM) than expected, because BP-Impute assumes that there is only one recombination event within an ambiguous interval and it is possible that in some intervals there are multiple recombination events. R/QTL (Broman *et al.* 2003; Broman and Sen 2009) imputes the missing regions via the Viterbi algorithm (method 2), and may introduce additional breakpoints absent in the BP-Impute datasets. This results in the larger average two-point genetic maps for R/QTL, at 1554.5 cM (SD ± 161.7 cM). Mapmaker/EXP3.0 (Lincoln *et al.* 1993), with multipoint genetic maps (method 1), produced the largest maps at 2177.0 cM (SD ± 352.8 cM) without error detection (Lincoln and Lander 1992). Method 1 was shorter than method 2 and comparable to method 3, at 1494.2 cM (SD ± 218.2 cM) with error detection (Figure 5). The large disparity between the error-corrected datasets indicates that many of the variants within the ambiguous and unimputed regions, if not checked for accuracy, may induce erroneous recombination results.

The joint dataset has a smaller genetic map size (1348.7 cM) than the average of the 10 populations (1430.8 cM) (Figure 5 and Tables S2 and S3 in File S1). Since the joint dataset was imputed with R/QTL argmax (Broman *et al.* 2003; Broman and Sen 2009), residual heterozygosity was imputed as homozygous. This results in false homozygous genotypes and the smaller genetic map. Therefore, the joint dataset has limitations in genotype accuracy and the number of detected recombination events that the individual population datasets do not possess. However, because the joint dataset contains genotypes from all populations in a single flat text file, it facilitates an initial joint QTL mapping survey.

The greatest strength of using the BP-Impute dataset is the preservation of residual heterozygosity. Unlike R/QTL argmax, which imputes heterozygous regions as homozygous, LB-Impute and BP-Impute parse heterozygous regions and impute their breakpoints. This could be significant in the rice NAM datasets, as many NAM lines had residual heterozygosity (Table 1). Including the residual heterozygosity in the genetic map calculations allowed comparison with methods 1 and 2, which assume infinite number of selfing generations in the RILs, and increased the average map size from 1210.2 to 1430.8 cM (Figure 5).

Should the 3000 Rice Genomes Project data (Rice Genomes Project 2014) have been available during the creation of these NAM lines, an improved experimental design would have recruited parental lines more representative of *tropical japonica* diversity and with less *indica* admixture. A resource such as the 3000 Rice Genomes Project is essential to ensure that a panel of parental lines fully maximizes the genetic diversity found in a phylogenetic group of rice. Our PCA (Figure 3) indicates that some of the diversity donors (CT8556-37-1-3-1-M and CT10035-26-4-2-M in particular) appear to have been heavily admixed with *indica*. As long as the admixed lines harbor traits of interest also present in the other, unadmixed parentals, the admixed lines warrant inclusion in the founder panel to facilitate joint mapping analysis. However, if the primary goal is to create a diversity archive as well as a mapping population, admixture between the diversity donors and the common, reference parent should be avoided.

The 10 populations described in this study are well suited for detailed study of segregation distortion, on an individual or joint population basis. As an example, we focused on two regions on chromosome 3. Chromosome 3 is known to contain a multitude of segregation distortion loci, especially in the context of *indica* × *japonica* crosses—which are known to segregate for multiple sterility genes—such as *ga2*, *ga3*, and *S34* (Lin *et al.* 1992; Harushima *et al.* 1996; Xu *et al.* 1997; Lu *et al.* 2000; Matsushita *et al.* 2003; Zhang *et al.* 2005; Wu *et al.* 2010; Kim *et al.* 2014). In many *indica* × *japonica* populations, such as F_2_ (Harushima *et al.* 1996; Xu *et al.* 1997), BC (Xu *et al.* 1997; Kim *et al.* 2014), and RIL (Xu *et al.* 1997), the *indica* allele tends to be favored in regions of segregation distortion. In our study, we showed that the majority of segregation distortion loci favor the IR64 *indica* allele in multiple populations (Figure S1 and Table S1 in File S1), whereas there were *japonica*-favored regions on chromosomes 1, 4, and 6 that favored one population. In order to demonstrate the ability of the NAM populations to identify gene candidates for distortion factors, both within an individual population and jointly across several, we further inspected two loci on chromosome 3. Gene candidates identified by this study may contribute to the architecture of previously known segregation distortion on chromosome 3.

The first population-specific segregation distortion peak for IR64 × CT10045-5-5-M-1 was found at 7.6 Mb. A second, shared segregation distortion region among six populations was found at 13.3 Mb on chromosome 3 (Figure 4). The 1.5-χ^2^ support intervals for these two regions from population-specific and pooled χ^2^ tests were 94,269 bp (population-specific, at Chr3:7.6 Mb) and 16,015 (shared, at Chr3:13.3 Mb). Under the assumption that there is only one shared distortion factor in this interval, we examined the support interval for the pooled χ^2^ values. In the vicinity of 13.3 Mb, the pooled 1.5-χ^2^ support interval included two genes, one of which, LOC_Os03g23030, is homologous to the maize pollen gene *aberrant pollen 1* (Xu and Dooner 2006). The close proximity of this pollen gene homolog to the peak distortion signal makes it a gene of interest for further examination of a potential role in gametic selection or sterility in rice.

For the IR64 × CT10045-5-5-M-1-specific interval at 7.6 Mb, there are 16 genes within the 1.5-χ^2^ support interval. At least two are of special interest for their role in pollen tube formation. One, LOC_Os03g13860, is a ras-related protein. Ras-related proteins are in the family of Rab GTP-binding proteins (Cheung and Wu 2008), and the Rab GTPase *RabA4d* in *Arabidopsis* regulates pollen tube growth (Szumlanski and Nielsen 2009). A second gene in this interval is the Actin Depolymerizing Factor (*ADF*) LOC_Os03g13950. The actin cytoskeleton is a crucial component of pollen tube elongation (Yokota and Shimmen 2006).

The NAM datasets also proved useful tool for complex trait mapping. We examined the QTL architecture of the first 15 Mb of chromosome 3, for the trait days to heading, both in individual populations and jointly (Figure 7 and Figure S4 in File S1). The short arm of chromosome 3 is especially rich with photoperiod genes and days to heading QTL (Chen *et al.* 2014; Lee and An 2015a,b). Four genes in particular have been well described. The CCCH-type zinc finger protein, early heading date 4 (Ehd4), has been shown to upregulate activity of the florigen genes *Ed3a* and *RFT1* (Gao *et al.* 2013). The MIKC-type MADS-box protein (OsMADS50) is a flowering activator, and interacts with the *OsGI*–*Hd1*–*Hd3a* flowering pathway (Lee *et al.* 2004). The *DNA-binding with one finger 12* gene (*OsDof12*) is a transcription factor that controls the expression of *Hd3a* (Li *et al.* 2009). *Rice phytochrome B* (*OsPhyB*), one of three phytochrome genes found in rice (Takano *et al.* 2005), has also been implicated in photoperiod monitoring and is also found in this interval. In our study, we examined population-specific and shared QTL peaks in the vicinity of these four genes.

Significant QTL peaks adjacent to three of the four known photoperiod genes in this region were identified. For one of those genes, *OsMADS50*, we further investigated whether there may be other genes of interest in this region besides *OsMADS50*. We calculated the support intervals for each individual population, and examined the region of their greatest consensus. When 1.5-*F* value support intervals were measured for each individual population, it appeared that there was a disjoint QTL structure in this region, with the interval of greatest consensus being an 85,226 bp region downstream of *OsMADS50*. This consensus support interval contains a protein receptor-like kinase with homology to *Arabidopsis* *CURVY1* (MSU rice BLAST: *e* score 10^−160^, top query coverage 96.32, top Id 38.57) implicated in flowering time control (Gachomo *et al.* 2014). When the 3-*F* value support intervals were used, an expanded consensus interval of 235,514 bp containing *OsMADS50* was identified. Notably, in a previous genome-wide association study of days to heading in elite tropical rice lines, a peak association signal was also detected 800 kb downstream from *OsMADS50* (Begum *et al.* 2015). The region surrounding *OsMADS50* may therefore contain additional gene candidates that have not yet been described.

*OsPhyB* did not reach significant QTL thresholds in joint analysis, and no population exhibited strong marginal effects. This lack of signal is interesting because of *OsPhyB’*s previous implication in flowering time control by repressing flowering under long day conditions (Takano *et al.* 2005; Jeong *et al.* 2007) and the gene’s polymorphism between the *tropical japonica* diversity donors and the *indica* common parent. Reasons for a lack of QTL signal may include both environmental and genetic factors, in addition to reduced detection power brought about by segregation distortion. The NAM lines were grown and phenotyped in Cali, Colombia, at a latitude of just 3.42° N, where days and nights are nearly equal all year round. This is likely to reduce the effect of long day flowering inhibition by *OsPhyB*. For genetic causes, nonadditive and epistatic effects have been shown to mask single QTL (Yamamoto *et al.* 2000). Finally, segregation distortion has been shown to reduce QTL detection power (Xu 2008), and *OsPhyB* exists in a region of segregation distortion on chromosome 3. In future studies of the NAM populations, phenotyping will occur in a variety of environments, and nonadditive genetic effects will be investigated to further explain the genetic basis of complex traits such as days to heading.

The genotypes and germplasm used in this study are freely available for use in research. All imputation software is available online. Any auxiliary scripts used to process the data are also available upon request. Parental, offspring, and breakpoint imputation used here have also been included as part of the latest release of MapDisto 2.0 (Lorieux 2012; Heffelfinger *et al.* 2017).

## Supplementary Material

Supplemental material is available online at www.g3journal.org/lookup/suppl/doi:10.1534/g3.117.041608/-/DC1.
