# Supplementary material for: Genetic Architecture of a Rice Nested Association Mapping Population
Source: G3 (Bethesda). 2017 Apr 24;7(6):1913–26. doi: 10.1534/g3.117.041608 (PMC5473768; doi:10.1534/g3.117.041608)
Supplement: Supplementary file 1 [file 1913File002.docx]

Supplementary Methods Note 1:

**Bioinformatics processing example commands and parameters**

**Read alignment and bam processing**

novoalign -d nipponbare_msu_7.0.nix -f ID204-ID152_A03_TTAGGC-ATCACG_L001_R1_001.fastq ID204-ID152_A03_TTAGGC-ATCACG_L001_R2_001.fastq -i PE 200,200 -o SAM | samtools-1.3/samtools view -bS -> ID204-ID152_A03_TTAGGC-ATCACG.bam;

novosort ID204-ID177_H09_GTTTCG-GTGGCC.bam ID177-H09_GTTTCG

-GTGGCC.bam > ID177-H09_GTTTCG-GTGGCC.bam;

java -jar picard-tools-2.0.1/picard.jar SortSam I= ID177-H09_GTTTCG-GTGG

CC.bam O=ID177-H09_GTTTCG-GT

GGCC.bam SO=coordinate;

java -jar picard-tools-2.0.1/picard.jar AddOrReplaceReadGroups INPUT=ID177-H09

_GTTTCG-GTGGCC.bam OUTPUT=

ID177-H09_GTTTCG-GTGGCC.bam RGID=group1 RGLB= lib1 RGPL=illumina RGPU=

unit1 RGSM=ID177-H09_GTTTCG-GTGGCC; java -jar picard-tools-2.0.1/picard.jar BuildBamIndex INPUT=ID177-H09_GTTTCG-GTGGCC.bam; java -jar GenomeAnalysisTK.jar -T RealignerTargetCreator -R nipponbare_msu_

7.0_sed_correct.fa –I ID177-H09_GTTTCG-GTGGCC.bam -o ID177-H09_GTTTCG-GTGGCC_realigned.list; java -jar GenomeAnalysisTK.jar -T IndelRealigner -R nipponbare_msu_7.0_sed_correct.fa -I ID177-H09_GTTTCG-GTGGCC.bam –targetIntervals ID177-H09_GTTTCG-GTGGCC_realigned.list -o ID177-H09_GTTTCG-GTGGCC.bam;

**GATK variant calling**

java -jar GenomeAnalysisTK.jar -T UnifiedGenotyper –R nipponbare_msu_7.0_sed_correct.fa -o IR64xAzucena_reseq_raw.vcf -I ID152bA01-P1_ATCACG-ATCACG.bam

Note: For WGS parental analysis, the same commands we used as above, but with the following parameter changes:

Novoalign, i=PE 101,101

Variant filtering:

java -jar VarFilt.jar -f NAM_parentals_WGS_raw.vcf -QD 2 -MAC 2 -FS 60 -HS 10 -MQ 40 -MQRS -12.5 -RPRS -8 -samplefract 0.2 -hetcheck 0.01 0.99 -RTA -bamdir parental_mapped_sorted_realigned/ -o NAM_parentals_WGS_filtered.vcf

**Imputation and parental imputation filtering**

*Parental imputation*: java -jar LB-Impute.jar -method impute -f IR64xAzucena_reseq_filtered.vcf -o IR64xAzucena_reseq_filtered_parimpute.vcf -parentimpute -resolveconflicts -recombdist 10000000 -readerr 0.05 -genotypeerr 0.05 -window 7 -minsamples 5 -minfraction 0.5 -parents ID152bH10-P2_CGTACG-GTGGCC,ID152bH11-P2_GAGTGG-GTGGCC;

*Parental imputation filtering*: java -jar VarFilt.jar -f IR64xAzucena_reseq_filtered_parimpute.vcf -minparcov 1 -parfract 1 -parhom -pardiff -parents ID152bH10-P2_CGTACG-GTGGCC,ID152bH11-P2_GAGTGG-GTGGCC -o IR64xAzucena_reseq_filtered_parimpute_parfilt.vcf;

*Offspring imputation*: java -jar LB-Impute.jar -method impute -f IR64xAzucena_reseq_filtered_parimpute_parfilt.vcf -o IR64xAzucena_reseq_filtered_parimpute_parfilt_imputed.vcf -offspringimpute -recombdist 10000000 -readerr 0.05 -genotypeerr 0.05 -window 7 -dr -parents ID152bH10-P2_CGTACG-GTGGCC,ID152bH11-P2_GAGTGG-GTGGCC;

**BP-Impute**

Rscript bpimpute_v33_final.R IR64xAzucena_reseq_filtered_parimpute_parfilt_imputed_keep.vcf 43 ID152bH10.P2_CGTACG.GTGGCC ID152bH11.P2_GAGTGG.GTGGCC 0.1 trim yeshet;

**Assign genotypes**

Rscript assign_genotypes_v13.R IR64xAzucena_weighted_genos_trim_yeshet.txt IR64xAzucena_imputed_binary_trim_yeshet.txt;

Supplementary Methods Note 2:

**BP-Impute algorithm**

BP-Impute is a hidden Markov model method, like LB-Impute {Fragoso, 2016 #92}, but features a few key differences. A Markov chain is constructed from either end of an ambiguous breakpoint region. Each chain from either direction, however, is constrained to the hidden state of the last marker imputed by LB-Impute. BP-Impute works under the assumption that there is only one transition in parental state in the ambiguous interval. The initial probability of the high-confidence LB-Impute marker’s parental state is 1. Transition probabilities are calculated from the proportion of recombined lines within the missing interval, for the entire population. This is a rather naïve measure of transition probabilities that will be improved in future versions. The probability calculation for one marker, for one homozygous parental state, in a left to right Markov chain, is demonstrated in Equation 1:

| $P_{right}\left( {state}_{t}={parent}_{A} \right)=P\left( {state}_{t-1}={parent}_{A} \right)*\left( 1-P\left( {recombination}_{Interval} \right) \right)* P\left( homozygous emission \right\vert{state}_{t}={parent}_{A})$ | Eq. 1 |
| --- | --- |

Sequenced (yet unimputed) markers within the ambiguous regions may be incorporated into the model in two ways. One way is to view the read depth as an emission from the constrained parental state. This may include valuable additional information to fine tune the breakpoint, as aligned reads may greatly support one parental state over the other. For this assumption, we use the same binomial emission model as with LB-Impute. Second, sequenced markers could be assumed to have high confidence genotypes, just as with the LB-Impute imputed marker set. These markers then also serve as “anchors” to the Markov chains. This is particularly useful at the distal ends of chromosomes, which LB-Impute often leaves unimputed.

The genotype probabilities from each chain, after being normalized to sum at 1, may then be used to weight each parental genotype, and a weighted average genotype is produced. The probabilities are then divided by 2 so that the maximum value is 1. For assigning discrete genotypes to the probabilities, a separate R script is used (assign_genotypes.R) that employs least squares to identify the breakpoint. Then, markers on either side of the breakpoint are assigned the proper genotype. If the probabilities are exactly halfway in between two parental states, the breakpoint is randomly assigned.


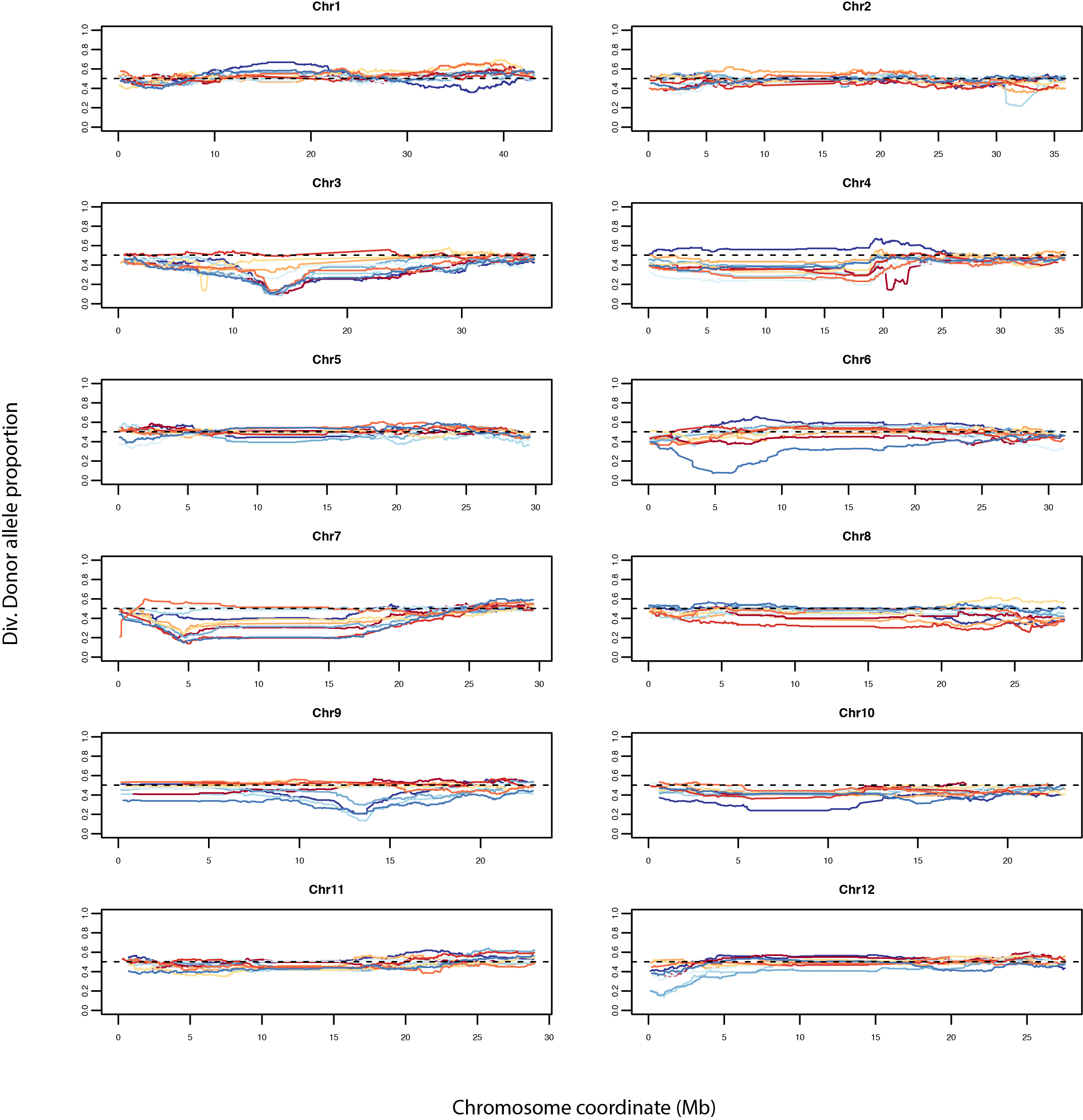


**Supplementary Figure 1** Parental contribution in the NAMs, all chromosomes

The proportion of diversity donor alleles at each marker, for every population of NAMs. Each color represents a different population; color coding is consistent with the figures in the main body of the text. The horizontal dotted line is at 0.5, the 1:1 expected segregation ratio of parental alleles in a recombinant inbred line.

| Distortion locus | Populations | Diversity donor proportion extremity^1^ | Chi-square statistic^2^ | -log10 *P* value^2^ |
| --- | --- | --- | --- | --- |
| Chr3:7591055 | 1 | 0.13 | 195.14 | 43.62 |
| Chr3:13289973 | 6 | 0.10 | 1363.51 | 284.00 |
| Chr6:4818659 | 1 | 0.07 | 261.38 | 58.07 |
| Chr7:4695065 | 6 | 0.15 | 814.35 | 165.86 |
| Chr9:13566353 | 4 | 0.14 | 523.83 | 107.27 |

^1^Genotyped markers before linear interpolation

^2^Pooled value if populations > 1

**Supplementary Table 1** Loci of segregation distortion in the direction of the IR64 allele

**Supplementary Figure 2** Distribution of the number of detectable recombination events in the 10 populations

The number of transitions between parental states (homozygous IR64, homozygous diversity donor, heterozygous) were counted for each line and plotted as a distribution for each population. The color key is the same as the other figures in the main body of the test. Anova suggests the 10 population means differ significantly, with an *F* value of 4.5 and a *P* value of 6.8 x 10^-6^. The gray population is the sum of all NAM lines treated as one greater population. The NAM average is 18.9 recombination events, with a standard deviation of 10.9.

| Diversity donor of population | Chr1 | Chr2 | Chr3 | Chr4 | Chr5 | Chr6 | Chr7 | Chr8 | Chr9 | Chr10 | Chr11 | Chr12 | Total size |
| --- | --- | --- | --- | --- | --- | --- | --- | --- | --- | --- | --- | --- | --- |
| Azucena | 187.8 | 150.3 | 172.1 | 126.1 | 122.0 | 117.3 | 119.9 | 101.4 | 81.3 | 82.4 | 89.1 | 90.1 | 1439.9 |
| ITA164 | 179.2 | 160.6 | 169.4 | 121.0 | 110.7 | 114.1 | 105.3 | 109.8 | 85.4 | 78.0 | 96.0 | 88.0 | 1417.6 |
| CT10035-42-4-4-M | 198.3 | 162.3 | 175.7 | 117.9 | 120.7 | 122.1 | 121.0 | 111.6 | 86.8 | 85.2 | 98.3 | 85.9 | 1486.0 |
| CT10006-7-2-M-2 | 215.5 | 204.9 | 172.2 | 129.2 | 139.5 | 126.6 | 123.0 | 115.1 | 87.7 | 87.8 | 108.4 | 102.1 | 1612.0 |
| CT10037-56-6-M-M | 185.8 | 152.4 | 160.1 | 108.9 | 122.6 | 116.1 | 86.3 | 103.5 | 82.8 | 72.9 | 106.0 | 86.8 | 1384.1 |
| CT10045-5-5-M-1 | 200.4 | 162.1 | 166.5 | 133.7 | 123.3 | 130.0 | 118.5 | 109.3 | 83.4 | 76.6 | 89.9 | 96.8 | 1490.4 |
| CT10005-12-1-M-4 | 199.0 | 155.8 | 144.5 | 122.8 | 113.6 | 137.7 | 113.3 | 94.3 | 66.2 | 88.8 | 99.9 | 91.3 | 1427.3 |
| CT9998-41-12-M-4 | 197.0 | 154.0 | 163.9 | 123.6 | 118.8 | 127.8 | 127.6 | 98.2 | 82.1 | 79.4 | 92.4 | 85.4 | 1450.1 |
| CT8556-37-1-3-1-M | 173.9 | 152.5 | 150.1 | 115.6 | 111.9 | 114.5 | 119.6 | 108.3 | 57.7 | 72.3 | 105.8 | 62.7 | 1344.9 |
| CT10035-26-4-2-M | 162.6 | 129.6 | 160.7 | 127.7 | 98.9 | 109.0 | 96.1 | 85.0 | 84.9 | 43.3 | 84.0 | 74.1 | 1255.8 |
| Mean | 190.0 | 158.4 | 163.5 | 122.7 | 118.2 | 121.5 | 113.1 | 103.7 | 79.8 | 76.7 | 97.0 | 86.3 | 1430.8 |
| SD | 15.29 | 18.84 | 10.02 | 7.21 | 10.61 | 8.87 | 13.16 | 9.16 | 9.85 | 13.05 | 8.19 | 11.11 | 94.43 |

**Supplementary Table 2** Population genetic map sizes by chromosome, estimated by BP-Impute, including homozygous-heterozygous transitions, through two-point analysis.

| Chromosome | Size (cM) | Number of Markers | Density (Markers per cM)^1^ |
| --- | --- | --- | --- |
| 1 | 180.0 | 7264 | 0.025 |
| 2 | 149.4 | 5726 | 0.026 |
| 3 | 152.8 | 5580 | 0.027 |
| 4 | 115.8 | 4018 | 0.029 |
| 5 | 111.6 | 4141 | 0.027 |
| 6 | 114.3 | 3860 | 0.030 |
| 7 | 106.2 | 3579 | 0.030 |
| 8 | 98.2 | 3605 | 0.027 |
| 9 | 74.6 | 2645 | 0.028 |
| 10 | 72.2 | 2651 | 0.027 |
| 11 | 91.9 | 4053 | 0.023 |
| 12 | 81.7 | 2884 | 0.028 |
| Sum | 1348.7 | 50006 |  |

**Supplementary Table 3** Joint genetic map sizes by chromosome, measured with R/QTL through two-point analysis and the Martin-Hospital estimate of recombination per meiosis in RILs.

^1^Average density was 0.027 markers per cM

**Supplementary Figure 3** Distribution of days to heading in the 10 NAM populations

Days to heading is defined here as the number of days to the emergence of the rice inflorescences since sowing date. The color coding is consistent with the figures in the main body of the text. The global mean of days to heading, among all NAM lines, was 91.86 days, with a standard deviation of 6.69 days. The greatest mean days to heading, for an individual population, was IR64 x CT10035-26-4-2-M at 99.90 days. The fewest mean days to heading was IR64 x Azucena at 88.82 days. Anova suggested that differences between the population means were statistically significant, with an *F* value of 55.00 and a *P* value < 2.2 x 10^-16^.

**Supplementary Figure 4** *F* values for single marker regression of days to heading on the first 15 Mb of Chromosome 3

Each plot is from a different NAM population; the color coding is consistent with the figures in the main body of the text. The *x* axis is the first 15 Mb of chromosome 3, with vertical lines representing the positions of 4 known photoperiod control genes in this region, from left to right, *Ehd4*, *OsMADS50*, *OsDof12*, and *OsPhyB*. The *y* axis represents the *F* values on a linear scale, with the horizontal line as is the maximum *F* value found in any population (69.79, IR64 x Azucena).
